# Impact of rapid identification of positive blood cultures using the Verigene system on antibiotic prescriptions: A prospective study of community-onset bacteremia in a tertiary hospital in Japan

**DOI:** 10.1371/journal.pone.0181548

**Published:** 2017-07-24

**Authors:** Kayoko Hayakawa, Kazuhisa Mezaki, Masao Kobayakawa, Kei Yamamoto, Yoshikazu Mutoh, Motoyuki Tsuboi, Takehiro Hasimoto, Maki Nagamatsu, Satoshi Kutsuna, Nozomi Takeshita, Yuichi Katanami, Masahiro Ishikane, Norio Ohmagari

**Affiliations:** 1 Disease Control and Prevention Center, National Center for Global Health and Medicine, Tokyo, Japan; 2 Department of Clinical Laboratory, National Center for Global Health and Medicine, Tokyo, Japan; 3 Department of Gastroenterology, National Center for Global Health and Medicine, Tokyo, Japan; 4 Division of Global infectious diseases, Department of Infection and Epidemiology, Graduate School of Medicine, Tohoku University, Miyagi, Japan; University of British Columbia, CANADA

## Abstract

**Background:**

Rapid identification of positive blood cultures is important for initiation of optimal treatment in septic patients. Effects of automated, microarray-based rapid identification systems on antibiotic prescription against community-onset bacteremia (COB) remain unclear.

**Methods:**

We prospectively enrolled 177 patients with 185 COB episodes (occurring within 72 h of admission) over 17 months. Bacteremia episodes due to gram-positive bacteria (GP) and gram-negative bacteria (GN) in the same patient were counted separately. For GP bacteremia, patients with ≥2 sets of positive blood cultures were included. The primary study objective was evaluating the rates of antibiotic prescription changes within 2 days of rapid identification using the Verigene system.

**Results:**

Bacteremia due to GN and GP included 144/185 (77.8%) and 41/185 (22.2%) episodes, respectively. Antibiotic prescription changes occurred in 51/185 cases (27.6% [95%CI:21.3–34.6%]) after Verigene analysis and 70/185 cases (37.8% [30.8–45.2%]) after conventional identification and susceptibility testing. Prescription changes after Verigene identification were more frequent in GP (17/41[41.5%]) than in GN (34/144[23.5%]). Among bacteremia due to single pathogen targeted by Verigene test, bacterial identification agreement between the two tests was high (GP: 38/39[97.4%], GN: 116/116[100%]). The Verigene test correctly predicted targeted antimicrobial resistance. The durations between the initiation of incubation and reporting of the results for the Verigene system and conventional test was 28.3 h (IQR: 25.8–43.4 h) and 90.6 h (68.3–118.4 h), respectively. In only four of the seven episodes of COB in which two isolates were identified by conventional tests, the Verigene test correctly identified both organisms.

**Conclusion:**

We observed a high rate of antibiotic prescription changes after the Verigene test in a population with COB especially in GP. The Verigene test would be a useful tool in antimicrobial stewardship programs among patients with COB.

## Introduction

Rapid identification of positive blood cultures is important for the rapid initiation of optimal treatment in patients with bacteremia, increasing the opportunity for antimicrobial stewardship programs (ASPs) [1]. Among the variety of available rapid identification systems, the Verigene gram-positive and gram-negative blood culture nucleic acid test (BC-GP, BC-GN; Nanosphere Inc., Northbrook, IL, USA), an automated microarray-based rapid identification system, is able to identify multiple bacterial species and their resistance genes, and the microbiological and clinical utility and impact of automated systems have been reported [2–5]. Considering its ability to identify common resistance genes and some typical healthcare-associated bacteria (e.g., methicillin-resistant *Staphylococcus aureus* [MRSA], *Pseudomonas aeruginosa*, *Acinetobacter* spp., and *Serratia marcescens*), the use of the Verigene system is expected to have clinical impact, including effects on antimicrobial prescriptions against hospital-acquired bacteremia. In fact, a previous study showed that this system decreased the time to both effective and optimal antibiotic therapy by BC-GN with antimicrobial stewardship intervention in 79 out of 132 (59%) cases of hospital-acquired bacteremia [2].

Nosocomial acquisition of bacteremia has been shown to be independently associated with in-hospital mortality [6]. However, because resistant pathogens, particularly extended spectrum β-lactamase (ESBL)-producing *Escherichia coli*, have recently spread to the community and have caused bacteremia [7], and because there is a risk of morbidity due to the unknown duration of bacteremia in patients with community-onset bacteremia (COB) [8], COB is an important target of optimization for antibiotic treatment.

Accordingly, in this study, we aimed to identify the effects of BC-GP and BC-GN tests on antibiotic prescriptions against COB.

## Methods

### Study design and patients

We conducted a prospective cohort study between August 2014 and December 2015 at the National Center for Global Health and Medicine (NCGM), Tokyo, Japan. NCGM has more than 780 inpatient beds and serves as a tertiary referral hospital for metropolitan Tokyo.

The primary study objective was to evaluate the rates of antibiotic changes within 2 days of rapid microbiological identification by the Verigene system (i.e., the same day when the Verigene test result became available and the next day until 12:00 AM) among patients who developed COB. Antibiotic changes included switching to other antibiotics and discontinuation of initial antibiotics. The rates of antibiotic changes within 2 days (i.e., the same day when the conventional culture result became available and the next day until 12:00 AM) of conventional test (conventional microbiological identification and susceptibility test) were also obtained and were compared with those of the Verigene system.

COB was defined as bacteremia occurring within 72 h of admission. For GP bacteremia, only patients with two or more sets of positive blood cultures were included to exclude contamination episodes. Only the first episode of bacteremia for each patient during the study period was included. Episodes of bacteremia due to gram-positive bacteria (GP) and gram-negative bacteria (GN) in the same patient were counted separately. De-escalation was defined according to a previously described ranking of β-lactams [9]. In addition, reducing the numbers of antibiotics (e.g., vancomycin and ceftriaxone to ceftriaxone), switching from third- or fourth-generation cephalosporins to first-generation cephalosporins, switching from first-generation cephalosporins to penicillin (including aminopenicillin) and switching from aminopenicillin to penicillin G were also considered de-escalation. This study was approved by the Human Research Ethics Committee of NCGM (NCGM-G-001576-02) prior to its initiation, and written consent was obtained from every subject.

### Microbiological analysis

Microarray-based rapid identification of positive blood culture was conducted using the Verigene system within 24 h after blood cultures became positive. The Verigene test was not performed at night owing to the inability to obtain consent and the limited daytime hours of the microbiology department. According to the Gram staining results of positive blood cultures, a BC-GP or BC-GN panel was chosen for further identification by the Verigene system. BC-GP or BC-GN targets were reported if positive. If the BC-GP or BC-GN panel was negative for all panel targets, results were reported as not detected. Results were reported at patients’ medical record as well as direct communication with investigators (i.e. infectious diseases [ID] physician) to the primary team. As soon as Verigene test results became available, the microbiology department notified the investigators, who communicated with the primary team immediately over the phone and provided advice regarding the antibiotic regimen based on these results. The final decision for antibiotic selection was made by the primary team, and adherence to the advice provided by investigators was not mandatory. Patients with blood cultures that became positive over holidays were not included except special holidays when investigators were available for the direct communication with primary team. Conventional bacterial identification and susceptibilities to the predefined antimicrobials were determined using an automated broth microdilution system (MicroScan WalkAway; Beckman Coulter Inc., Japan) and in accordance with the Clinical and Laboratory Standard Institutions criteria (M100-S22) [10]. The microbiological analysis data were also compared between the two methods.

### Data collection

Data on the following parameters were retrieved from the medical charts and microbiological reports: patient demographics, results of Verigene BC-GP or BC-GN panels, microbiological identification and susceptibility results, and antibiotic regimens after hospitalization until 2 days after conventional microbiological identification and susceptibility.

### Statistical analysis

The primary study objective was to evaluate the rates of antibiotic prescription changes within 2 days of rapid identification using the Verigene system. On the basis of a previous study [11], the possible rate of antibiotic changes after rapid identification by the Verigene system was estimated as 35%. Using EZR, (Saitama Medical Center, Jichi Medical University, Saitama, Japan), we calculated that a sample size of 179 cases would produce a two-sided 95% confidence interval with a width equal to 0.14 when the sample proportion is 0.35. Bivariate analyses were performed using Fisher’s exact test and chi-square tests (categorical variables) or Mann-Whitney U tests (continuous variables). Two-sided *p* values of less than 0.05 were considered statistically significant. These analyses were performed using SPSS software (version 20). The confidence interval for rates of antibiotic change and concordance rates was obtained using EZR (Saitama Medical Center, Jichi Medical University, Saitama, Japan).

## Results

During the study period, 185 episodes of COB were identified in 177 patients.

**Fig 1:**
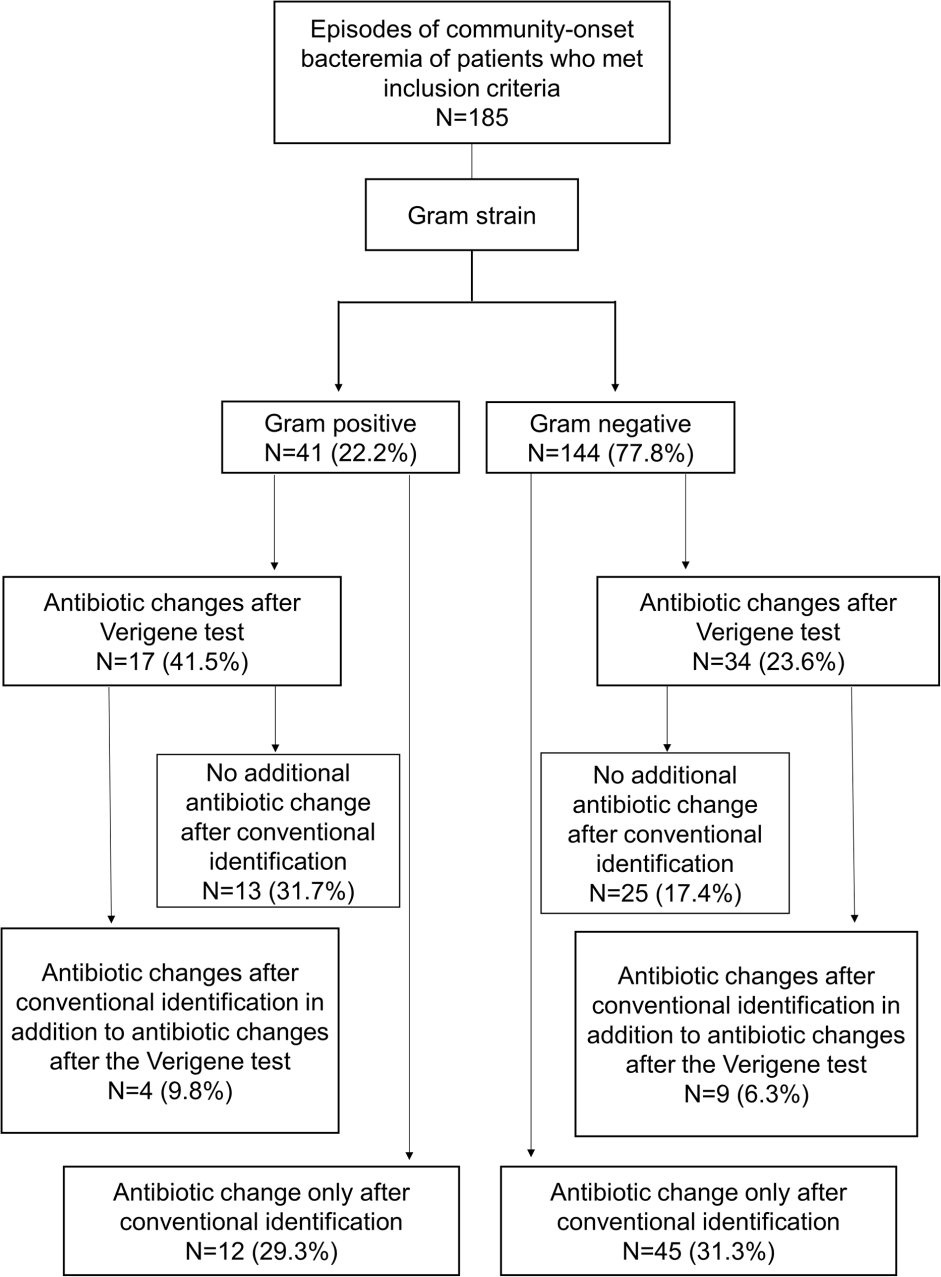
Flowchart of each test and subsequent antibiotic changes.

Bacteremia due to GN and GP included 144/185 (77.8%) and 41/185 (22.2%) episodes, respectively. The median age of the patients was 72 years (interquartile range [IQR]: 61–81 years), and 103 (58.2%) were male. A flowchart of each test and the subsequent antibiotic changes is provided in Fig 1. Antibiotic prescription changes occurred in 51/185 cases (27.6% [95% confidence interval: 21.3–34.6%]) after the Verigene test and 70/185 cases (37.8% [30.8–45.2%]) after conventional identification and susceptibility tests (Table 1). Following changes based on Verigene tests, there were no subsequent antibiotic changes based on conventional test results in 38/185 cases (20.5%) (GP: 13/41 [31.7%], GN: 25/144 [17.4%]) (31.7%). Prescription changes after Verigene-based identification were more frequent for GP (n = 17/41, 41.5%) than GN (n = 34/144, 23.5%; *p* = 0.024). Verigene tests resulted in de-escalation to narrower antibiotics in 36 (19.5%) episodes. De-escalation after Verigene tests was more common in GP (n = 13/41, 31.7%) than in GN (n = 23/144 16%; *p* = 0.02). The most common pattern of de-escalation in GP was the discontinuation of vancomycin (n = 6; 5 episodes of methicillin-sensitive *Staphylococcus aureus*, 1 episode of *Streptococcus agalactiae*), followed by changes to narrow-spectrum antibiotics (n = 5; 2 episodes of methicillin-sensitive *S*. *aureus* [ceftriaxone and meropenem to cefazolin], 1 episode of *Streptococcus pyogenes* [ampicillin to penicillin G], 1 episode of *S*. *pneumoniae* [piperacillin and tazobactam to ceftriaxone], and 1 episode of *Streptococcus* spp. [cefazolin to ampicillin]).

**Table 1:** Comparison of the rates of antibiotic changes after each test^A^.

Verigene test			
	Total (n = 185)	GP (n = 41)	GN (n = 144)
**Antibiotic changes**	51 (27.6%) [21.3–34.6%]	17 (41.5%) [26.3–57.9%]	34 (23.6%) [16.9–31.4%]
De-escalation to narrower-spectrum effective antibiotics	36 (19.5%) [14–25.9%]	13 (31.7%) [18.1–48.1%]	23 (16%) [10.4–23%]
Switching to effective antibiotics from ineffective antibiotics^B^	10 (5.4%) [2.6–9.7%]	3 (7.3%) [1.5–19.9%]	7 (4.9%) [2–9.8%]
**Conventional test**			
**Antibiotic change only after conventional identification**	57 (30.8%) [24.2–38%]	12 (29.3%) [16.1–45.5%]	45 (31.3%) [23.8–39.5%]
De-escalation to narrower-spectrum effective antibiotics	41 (22.2%) [16.4–28.8%]	9 (22%) [10.6–37.6%]	32 (22.2%) [15.7–29.9%]
Switching to effective antibiotics from ineffective antibiotics	1 (0.5%) [0–3%]	0 [0–3%]	1 (1.9%) [0–3.8%]
**Antibiotic changes after conventional identification in addition to antibiotic changes after the Verigene test**	13^C^ (7%) [3.8–11.7%]	4 (9.8%) [2.7–23.1%]	9 (6.3%) [2.9–11.5%]
De-escalation to narrower-spectrum effective antibiotics	11^D^ (5.9%) [3–10.4%]	3 (7.3%) [1.5–19.9%]	8 (5.6%) [2.4–10.7%]
Switching to effective antibiotics from ineffective antibiotics	0 [0–1.6%]	0 [0–3%]	0 [0–2.1%]

Note. Values indicate number (%) [95% confidence interval], unless otherwise indicated.

^A^ The rates of antibiotic change within 2 days of rapid identification by the Verigene system and conventional identification and susceptibility tests.

^B^ Including two episodes in which antibiotics were initiated after the Verigene test results became available.

^C^ Including three episodes (two GPC and one GNR) in which changes were difficult to attribute to either method due to the short period of notification of differences in results.

^D^ Including two episodes (one GPC and one GNR) in which changes were difficult to attribute to either method due to the short period of notification of difference in results.

Abbreviations. GP, gram-positive organisms; GPC, gram-positive cocci; GN, gram-negative organisms; GNR, gram-negative rods; NA, not available.

The most common pattern of de-escalation in GN was a switch from antipseudomonal antibiotics (e.g., carbapenem, cefepime, and piperacillin/tazobactam) to non-antipseudomonal antibiotics (e.g., ceftriaxone, cefmetazole, and cefazolin) (n = 12; 10 episodes of *E*. *coli* and 2 episodes of *K*. *pneumoniae* bactermia).

In 10 (5.4%) episodes, Verigene tests enabled rapid initiation of effective antibiotics switched from ineffective antibiotics; this was much more frequent than that for the conventional test (n = 1, 0.5%; *P* = 0.006). CTX-M-positive *E*. *coli* was identified in 4 cases, and antimicrobial regimens were changed from other beta-lactams to carbapenems in 3 cases and to piperacillin/tazobactam in 1 case. The rapid identification of *Enterococcus faecalis* (n = 1), *Enterococcus faecium* (n = 1), MRSA (n = 1), and *Pseudomonas aeruginosa* (n = 1) led to the prompt modification of antibiotics as follows: a switch from cefazolin to ampicillin (*E*. *faecalis*), the addition of vancomycin (*E*. *faecium*), a switch from cefazolin to vancomycin (MRSA), and a switch from ceftriaxone to piperacillin (*P*. *aeruginosa*). In two cases (*Escherichia coli* [n = 1], *Klebsiella pneumoniae* [n = 1]), empirical antibiotics were not initiated by the primary team, and effective antibiotics (3^rd^ generation cephalosporins) were started immediately after the Verigene test results became available.

Among 70 (37.8%) out of 185 antibiotic changes after conventional tests, 57 (30.8%) changes occurred only after conventional test results became available. Thirteen (7%) changes after conventional tests were additional antibiotic changes made after Verigene tests, including three cases (two gram-positive cocci [GPC], one gram-negative rod [GNR]) that were difficult to attribute to either method due to the short period of notification of differences in results.

Overall, bacterial identification agreement between the Verigene test and conventional test was very high when single bacteria targeted by Verigene test were isolated from blood culture bottles (Table 2). One isolate that was identified as *Staphylococcus epidermidis* by the conventional test was identified as coagulase-negative *Staphylococci* by Verigene BC-GP. Organisms that were not targeted by the Verigene system caused 21 episodes of COB, including *Salmonella* spp. (n = 13, including five *Salmonella* Paratyphi and four *Salmonella* Typhi), *Bacteroides* spp. (n = 3, including one *B*. *fragilis*, one *B*. *thetaiotaomicron*, and one *B*. *vulgatus*), *Aeromonas hydrophila* (n = 2), *Fusobacterium mortiferum* (n = 1), *Enterococcus gallinarum* (n = 1), and *Escherichia fergusonii* (n = 1). As expected, none of these isolates were identified by Verigene tests, except one *Escherichia fergusonii*, which was misidentified as *Escherichia coli* by Verigene BC-GN. In one COB episode, initial Gram staining falsely indicated GNR, and Verigene BC-GN was conducted, which resulted in no detection of any organism. Subsequently, conventional test results identified *Streptococcus pneumoniae*.

**Table 2:** Performance of Verigene BC-GP and BC-GN for bacteremia evaluation*.

Gram-positive	Correctly identified/total	Gram-negative	Correctly identified/total
*Staphylococcus aureus*	13/13 (100%) [79.4–100%]	*Serratia marcescens*	2/2 (100%) [22.4–100%]
Staphylococcus epidermidis**	1/2 (50%) [1.3–98.7%]	*Pseudomonas aeruginosa*	6/6 (100%) [60.7–100%]
Coagulase-negative *Staphylococci*	3/3 (100%) [36.8–100%]	*Klebsiella oxytoca*	4/4 (100%) [47.3–100%]
*Enterococcus faecalis*	3/3 (100%) [36.8–100%]	*Klebsiella pneumoniae*	23/23 (100%) [87.8–100%]
*Enterococcus faecium*	1/1 (100%) [5–100%]	*Escherichia coli*	73/73 (100%) [96–100%]
*Streptococcus anginosus* group	4/4 (100%) [47.3–100%]	*Enterobacter* spp.	4/4 (100%) [47.3–100%]
*Streptococcus pneumoniae*	1/1 (100%) [5–100%]	*Citrobacter* spp.	2/2 (100%) [22.4–100%]
*Streptococcus pyogenes*	2/2 (100%) [22.4–100%]	*Acinetobacter* spp.	2/2 (100%) [22.4–100%]
*Streptococcus agalactiae*	1/1 (100%) [5–100%]		
*Streptococcus* spp.	9/9 (100%) [71.7–100%]		

Note. Values indicate number (%) [95% confidence interval], unless otherwise indicated.

* Excluding episodes where multiple organisms were identified for each GP or GN test.

** One isolate was detected as coagulase-negative *Staphylococci*.

The performance of Verigene BC-GP and BC-GN in the identification of multiple organisms is summarized in Table 3. There were seven episodes of COB in which two isolates were identified by the conventional tests. With the exception of three episodes of COB as *E*. *coli* and *Klebsiella pneumoniae* and one episode of *K*. *pneumoniae* and *Citrobacter freundii*, in which both organisms were correctly identified by Verigene BC-GN, Verigene tests were unable to identify 1–2 organisms in each of these episodes. In one episode, two isolates (*K*. *pneumoniae* and *Enterobacter* spp.) were identified by verigene BC-GN; however, only *K*. *pneumoniae* was identified by the conventional test. Overall, the Verigene test correctly identified pathogens in 158 (85.4%) of 185 COB episodes.

**Table 3:** Performance of Verigene BC-GP and BC-GN in multiple organism identification^A^.

Conventional test results	Verigene test results
Coagulase-negative *Staphylococci* and *Staphylococcus aureus*	Coagulase-negative *Staphylococci*
*Klebsiella pneumoniae* and *Aeromonas hydrophila*	Not detected
*Klebsiella pneumoniae* and *Citrobacter freundii*	*Klebsiella pneumoniae* and *Citrobacter spp*.
*Escherichia coli* and *Klebsiella pneumoniae* ^B^	*Escherichia coli* and *Klebsiella pneumoniae*
*Citrobacter freundii* and *Enterobacter cloacae*	*Citrobacter* spp.
*Klebsiella pneumoniae*	*Klebsiella pneumoniae* and *Enterobacter* spp.

^A^ Each result included one episode unless otherwise stated.

^B^ Three episodes. In all cases, both detected.

The performance of Verigene BC-GP and BC-GN in resistance detection is presented in Table 4. The Verigene test correctly predicted all the targeted antimicrobial resistance identified by the conventional test. BC-GP detected two *mecA*-positive *Staphylococcus aureus* and one *mecA*-positive *S*. *epidermidis*, which were subsequently identified as MRSA and methicillin-resistant *S*. *epidermidis*, respectively, by the conventional tests. BC-GN detected 13 CTX-M-positive *E*. *coli* and one CTX-M-positive *K*. *pneumoniae*, which were subsequently identified as ESBL-producing *E*. *coli* (ESBL-*E*. *coli*) and ESBL-producing *K*. *pneumoniae*, respectively, by the conventional tests. No isolate, other than 14 GNRs (*E*. *coli* [n = 13] and *K*. *pneumoniae* [n = 1]) listed in Table 4, had elevated MIC values for 3^rd^ generation cephalosporins requiring further screening tests for ESBL production by conventional test methods (i.e., all *E*. *coli* [n = 60], *K*. *pneumoniae* [n = 22], and *K*. *oxytoca* [n = 4] had ceftriaxone MIC of 1 μg/mL). In other words, all ESBL-producing organisms isolated by conventional tests during the study period had CTX-M according to the Verigene test. No vancomycin-resistant enterococci (VRE) or carbapenemase-producing organisms were identified from patients with COB during the study period. The times from the initiation of incubation and reporting of the results for the Verigene system and conventional test were 28.3 h (IQR: 25.8–43.4 h) and 90.6 h (68.3–118.4 h), respectively. The elapsed times between the positive blood culture results and the reporting of results for the Verigene system and conventional tests were 4.3 h (IQR: 3.3–5.5) and 49 h (48–96 h), respectively.

**Table 4:** Performance of Verigene BC-GP and BC-GN in resistance detection.

Conventional test results	Verigene test results	Concordance rate (95% confidence interval)
MRSA (n = 2)	*mecA* (+) *Staphylococcus aureus* (n = 2)	100% (22.4–100%)
Methicillin-resistant *Staphylococcus epidermidis* (n = 1)	*mecA* (+) *Staphylococcus epidermidis* (n = 1)	100% (5–100%)
ESBL-producing *Escherichia coli* (n = 13)	CTX-M (+) *Escherichia coli* (n = 13)	100% (79.4–100%)
ESBL-producing *Klebsiella pneumoniae* (n = 1)	CTX-M (+) *Klebsiella pneumoniae* (n = 1)	100% (5–100%)

Abbreviations. ESBL, extended-spectrum β-lactamase; MRSA, methicillin-resistant *Staphylococcus aureus*.

## Discussion

To the best of our knowledge, this is the first study to identify the utility of Verigene BC-GP and BC-GN for patients with COB. Even for COB in which drug-resistant pathogens were expected to be less prominent than hospital-acquired bacteremia, the use of the Verigene system resulted in antibiotic changes in 51 (27.6%) out of 185 episodes, which included 36 (19.5%) episodes of de-escalation. Considering that Verigene BC-GP was able to provide information on key factors to consider anti-Gram-positive coverage (e.g., bacterial species and resistant genes, such as *mecA* and *vanA*), it is not surprising that antibiotic changes, particularly de-escalation, occurred more often in COB due to GP. Compared with GP, it was more difficult to narrow the antibiotic regimen against GN because Verigene BC-GN was not able to predict resistance profiles other than CTX-M-type ESBLs and carbapenemase-producing organisms, such as quinolone and/or aminoglycoside resistance, or detailed susceptibility patterns for beta-lactams.

Importantly, in 10 (5.4%) episodes, changes from ineffective to effective antibiotics were made after Verigene tests before conventional test results became available. Among these episodes, CTX-M-positive *E*. *coli* was most frequently identified (n = 4). As in other regions worldwide, ESBL-*E*. *coli* have spread to the community in Japan, where community-associated ESBL-*E*. *coli* infections have been reported to be account for a quarter of all ESBL-*E*. *coli* infections [11]. In the current study, 13 (17.1%) out of 76 COB due to *E*. *coli* were caused by CTX-M-ESBL-*E*. *coli*. In contrast, only one episode of MRSA was detected by Verigene BC-GP, which resulted in changing to effective antibiotics.

Previous studies have reported that the use of Verigene BC-GP and BC-GN resulted in quicker organism identification, shorter length of stay in the intensive care unit, shorter duration to effective and optimal therapy, lower costs, and lower mortality [2,4,5]. In this study, we used simple outcomes to measure the clinical impact of Verigene tests, i.e., the rate of antibiotic change within 2 days of rapid microbiological identification by the Verigene system. Changes in antibiotics after obtaining Verigene test results would not occur if only the conventional method had been used, clearly showing the impact of Verigene tests on COB.

However, our results indicated that caution is needed when implementing Verigene tests. First, Verigene relies on an accurate Gram stain results to set up the correct panel. In this study, one episode of COB due to *Streptococcus pneumoniae* was falsely identified as COB due to GNR, which resulted in the lack of detection by Verigene BC-GP. Second, Verigene test performance for the correct identification of multiple targeted organisms isolated from the same blood culture bottle was suboptimal, as presented in Table 3. This disagreement between the conventional test and Verigene test for polymicrobial blood cultures is consistent with the results of multiple previous studies, and this has been attributed to an insufficient quantity of targeted bacteria in the cultures [12,13].

A major limitation of this study was the lack of a control period during which the Verigene system was not used; thus, comparative analyses of data obtained before and after Verigene tests were not possible. Gram stain results based on morphologies alone might have contributed to some changes observed in this study. Antibiotic changes owing to the detection of the presence or absence of resistant genes such as *mecA* and CTX-M, differentiation of species (e.g. *E*. *faecalis* and *E*. *faecium*) could only be possible by the results of Verigene tests. Differentiation of *P*. *aeruginosa* from other Gram-negative rods by Verigene tests might also have contributed to the more appropriate antibiotic selections, as differentiation based on morphological characteristics is not always accurate [14]. In our study setting, in which Verigene tests were not conducted at night, might have resulted in an underestimation of the positive impacts of Verigene tests on the rapid identification of causative pathogens of bacteremia.

In this study, we included only patients with at least two sets of positive blood cultures to exclude contamination of GP bacteremia, and to maximize the inclusion of patients with bacteremia due to “true infection.” The inclusion of patients with one set of positive blood cultures for GP bacteremia may have revealed additional benefits of the rapid identification tests for pathogens of bacteremia, including the Verigene BC-GP test, such as an increase in the discontinuation of unnecessary antibiotics against COB [15] and reductions in the time in the intensive care unit, the total cost of care by the differentiation of coagulase-negative *Staphylococcus* and *S*. *aureus* [15,16], and the 30-day mortality by the differentiation of *E*. *faecium* and *E*. *faecalis* [17]. Additionally, this study was conducted in a tertiary hospital in Tokyo, Japan, where we constantly admit severely ill patients, including approximately 12000 emergently transported patients per year. Thus, this study included diverse bacterial species as causes of COB; however, bacteremia due to highly drug-resistant pathogens, such as carbapenem-resistant *Enterobacteriacea*, multidrug-resistant *Acinetobacter* spp., and VRE, were not included because they are not frequent causes of COB in this region. A recent meta-analyses revealed that molecular rapid diagnostic testing, including Verigene BC-GP and BC-GN, was associated with significant decreases in mortality risk only in the presence of an ASP [18]. In this study, decisions regarding antibiotic changes were made by our primary team. However, all Verigene test results were communicated directly to the primary team by the investigators (ID physicians), who then provided advice on possible antibiotic switches based on the Verigene results. The design involving ID physicians in the release of Verigene results may have also affected the results of this study.

In conclusion, we observed a high rate of antibiotic prescription changes after the Verigene test in a COB population. The Verigene system may be a strong asset for ASP in this population.
